# *ABRAXAS* (*FAM175A*) and Breast Cancer Susceptibility: No Evidence of Association in the Breast Cancer Family Registry

**DOI:** 10.1371/journal.pone.0156820

**Published:** 2016-06-07

**Authors:** Anne-Laure Renault, Fabienne Lesueur, Yan Coulombe, Stéphane Gobeil, Penny Soucy, Yosr Hamdi, Sylvie Desjardins, Florence Le Calvez-Kelm, Maxime Vallée, Catherine Voegele, The Breast Cancer Family Registry, John L. Hopper, Irene L. Andrulis, Melissa C. Southey, Esther M. John, Jean-Yves Masson, Sean V. Tavtigian, Jacques Simard

**Affiliations:** 1 Genomics Center, Centre Hospitalier Universitaire de Québec Research Center and Laval University, Quebec, Canada; 2 INSERM, U900, Mines ParisTech, Institut Curie, Paris, France; 3 Genome Stability Laboratory, Centre Hospitalier Universitaire de Québec Research Center, HDQ Pavillon, Oncology Axis, Quebec, Canada; 4 Genetic Cancer Susceptibility group, International Agency for Research on Cancer, Lyon, France; 5 Center for Epidemiology and Biostatistics, School of Population and Global Health, The University of Melbourne, Victoria, Australia; 6 Lunenfeld-Tanenbaum Research Institute, Mount Sinai Hospital, Toronto, Canada; 7 Department of Molecular Genetics, University of Toronto, Toronto, Canada; 8 Genetic Epidemiology Laboratory, The University of Melbourne, Victoria, Australia; 9 Cancer Prevention Institute of California, Fremont, United States of America; 10 Stanford University School of Medicine and Stanford Cancer Institute, Stanford, United States of America; 11 Department of Molecular Biology, Medical Biochemistry and Pathology, Laval University, Quebec, Canada; 12 Department of Oncological Sciences, University of Utah, Salt Lake City, United States of America; 13 Huntsman Cancer Institute, University of Utah, Salt Lake City, United States of America; CNR, ITALY

## Abstract

Approximately half of the familial aggregation of breast cancer remains unexplained. This proportion is less for early-onset disease where familial aggregation is greater, suggesting that other susceptibility genes remain to be discovered. The majority of known breast cancer susceptibility genes are involved in the DNA double-strand break repair pathway. *ABRAXAS* is involved in this pathway and mutations in this gene impair BRCA1 recruitment to DNA damage foci and increase cell sensitivity to ionizing radiation. Moreover, a recurrent germline mutation was reported in Finnish high-risk breast cancer families. To determine if *ABRAXAS* could be a breast cancer susceptibility gene in other populations, we conducted a population-based case-control mutation screening study of the coding exons and exon/intron boundaries of *ABRAXAS* in the Breast Cancer Family Registry. In addition to the common variant p.Asp373Asn, sixteen distinct rare variants were identified. Although no significant difference in allele frequencies between cases and controls was observed for the identified variants, two variants, p.Gly39Val and p.Thr141Ile, were shown to diminish phosphorylation of gamma-H2AX in MCF7 human breast adenocarcinoma cells, an important biomarker of DNA double-strand breaks. Overall, likely damaging or neutral variants were evenly represented among cases and controls suggesting that rare variants in *ABRAXAS* may explain only a small proportion of hereditary breast cancer.

## Introduction

DNA damage induced by endogenous and exogenous genotoxic agents can promote genomic instability and directly lead to various diseases, particularly cancer. Functionally intact DNA double-strand break (DSB) repair machinery is essential for the maintenance of genomic integrity and stability. Mutations in genes coding for proteins involved in this pathway have been shown to cause chromosomal aberrations and defective cell cycle checkpoints, leading to tumor development [1]. Germline mutations in genes involved in homologous recombination DNA repair have been associated with breast cancer susceptibility. These include high-penetrance susceptibility genes such as *BRCA1* [2,3], *BRCA2* [4, 5] and *TP53* [6] and the moderate penetrance genes *CHEK2* [7, 8], *ATM* [9, 10], *BRIP1* [11], *PALB2* [12, 13] and *MRE11A*, *RAD50* and *NBN* [14]. However, despite technical progress and studies with greater statistical power, only approximately 35% of the familial relative risk of breast cancer is currently explained by the known high- and intermediate-risk genes, suggesting that other breast cancer susceptibility genes, possibly involved in the homologous recombination repair (HRR) pathway, remain to be discovered.

ABRAXAS (FAM175A) (MIM 611143; NM_139076.2) is a coiled-coil domain-containing protein that forms, along with Rap80, BRCC36, BRE and BABAM1, the A-Complex [15–17], which regulates the G2/M checkpoint and DNA-end resection during HRR [18–20]. Studies have reported that ABRAXAS-depleted cells have augmented levels of single-strand DNA and show increased binding of RPA and RAD51 proteins, indicative of increased resected DNA ends [20, 21]. Excessive DNA resection can lead to loss of genomic integrity. ABRAXAS also directly interacts with the BRCA1 BRCT (BRCA1 carboxyl-terminal) repeats through its phosphorylated SPxF motif [22–24] and contributes to BRCA1-dependent DNA damage responses by localizing BRCA1 to DNA damage foci [16]. Depletion of ABRAXAS impedes the recruitment of BRCA1 to DNA damage sites, leading to impairment of G2/M checkpoint control after ionizing radiation (IR) induction [25].

Further attesting to the critical role of ABRAXAS in cellular response to DNA damage and potential involvement in cancer susceptibility, Castillo et al. recently reported that both homozygous and heterozygous *Abraxas* knockout mice exhibited decreased survival and increased tumor incidence [26]. Analysis of gene expression levels in human tumors in the TCGA database revealed reduced *ABRAXAS* gene expression in numerous cancers, including breast cancer. This study also showed that gene copy number loss of the *ABRAXAS* locus at chromosome 4q21 is frequently found in ovarian and breast cancers and that this loss is well correlated with reduced *ABRAXAS* expression levels in these cancers [26].

Solyom et al. reported the screening of 125 Northern Finnish breast cancer families for coding region and splice-site *ABRAXAS* mutations [27]. Their study identified a novel germline mutation (p.Arg361Gln) in *ABRAXAS* in three of the families. Segregation analysis was performed in two of the mutation-positive families, showing co-segregation between the p.Arg361Gln mutation and breast cancer phenotype. The missense mutation is located in the nuclear localization sequence (NLS) of *ABRAXAS* and affects the nuclear localization of the protein. Consequently, this mutation reduces the formation of BRCA1 and Rap80 foci at DNA damage sites, leading to IR hypersensitivity of cells and partially impairing the G2/M checkpoint [27]. Another recent study, reporting the sequencing of several homologous recombination genes in 390 ovarian carcinomas, identified the loss-of-function *ABRAXAS* germline mutation, c.1106insG, in 2 subjects, representing 2% of identified deleterious mutations [28].

Based on the evidence that a germline *ABRAXAS* mutation is associated with an increased risk of breast cancer in the Finnish population and owing to the crucial role of this protein in DSB repair, we sought to estimate the frequencies and nature of rare *ABRAXAS* variants in a sample of women with early onset breast cancer (*N* = 1,332) and frequency matched controls (*N* = 1,123) from three population-based centers of the Breast Cancer Family Registry (BCFR) [29] by screening *ABRAXAS* exons and exon/intron boundaries. We then followed a similar *in silico*-driven analysis strategy to that previously proven successful to identify the intermediate penetrance susceptibility alleles in *ATM* [10], *CHEK2* [7], *XRCC2* [30], *MRE11*, *RAD50* and *NBN* [14].

## Materials and Methods

### Ethics statement

This study was approved by the Research Ethics Committee of the Centre Hospitalier Universitaire de Québec (Project 123.05.08 / MP-CHUQ-CHUL-08-009), the University of Utah Institutional Review Board (IRB), the Ethics committee of the International Agency for Research on Cancer (IARC) and the local IRBs of the BCFR centers: the Health Sciences Human Ethics Subcommittee of the University of Melbourne, Australia; the Institutional Review Board of the Cancer Prevention Institute of California; and the Research Ethics Board of Mount Sinai Hospital, Ontario, Canada, from which samples were received. All participants gave written informed consent.

### DNA samples

Subjects (*N* = 2,455) were selected from the population-based BCFR centers [29]. Women were recruited between 1995 and 2005 at three centers: the Cancer Prevention Institute of California (USA), the Cancer Care Ontario (Canada) and the University of Melbourne (Australia). Selection criteria for cases (*N* = 1,332) were: the age at breast cancer diagnosis (≤45 years), race/ethnicity, and grandparents’ country of origin consistent with the ethnic heritage, that is Caucasian, Latino, East Asian or African-American ancestry. Cases were matched with controls (*N* = 1,123) within each center according to the age at recruitment (± 10 years from the age at diagnosis) and race/ethnicity. However, because of limited availability of controls in some racial/ethnic and/or age groups, the matching was not always one-to-one in these groups (Table 1).

**Table 1:** Distribution of cases and controls by study center and by ethnicity in the BCFR.

Study center	Ethnic group	Cases (*N* = 1,332)	Controls (*N* = 1,123)	Total (*N* = 2455)
***BCFR Ontario***	*All*	*314*	*463*	*777*
	Caucasian	302	459	761
	East Asian	8	4	12
	Latino	4	0	4
	African-American Ancestry	0	0	0
***BCFR Northern California***	*All*	*421*	*136*	*557*
	Caucasian	0	0	0
	East Asian	177	54	231
	Latino	146	46	192
	African-American Ancestry	98	36	134
***BCFR Australia***	*All*	*597*	*524*	*1121*
	Caucasian	561	510	1071
	East Asian	28	13	41
	Latino	8	1	9
	African-American Ancestry	0	0	0

### Mutation screening

Mutation screening of the *ABRAXAS* gene was performed on Whole-Genome Amplified (WGA) DNA obtained by mixing 5 ng of amplified DNA from each of two independent WGA reactions. The 9 coding exons, exon-intron boundaries and part of the promoter region were screened by High Resolution Melting curve (HRM) analysis on a LightCycler 480 (Roche Applied Science, Indianapolis, USA), followed by direct Sanger sequencing of the samples for which an aberrant melting curve was indicative of the presence of a sequence variant (S1 Fig). All exons and part of the promoter region were amplified using specific primer pairs (S1 Table) in PCR reactions of 10 μl: each reaction contained 10 ng of WGA DNA, 5 μl of Master mix (Roche Applied Science, Indianapolis, USA), 1 μl of primers (0.5μM each) and 1.2 μL of MgCl_2_ (3.6μM). The cycling conditions were as follow: 95°C for 5 min, 40 cycles of DNA denaturation at 95°C for 10 sec, annealing for 45sec at temperatures depending on each primer pair (S1 Table), and elongation at 72°C for 14 sec. According to the manufacturer’s recommendations, the size of amplicons was 250 bp or less in order to optimize amplification specificity and variant detection sensitivity. Prior to HRM analysis, a melting step consisting of a denaturation step at 95°C for 1 min, and a cooling step at 40°C for 1 min was carried out for heteroduplex formation. Finally, the PCR products were heated from 65°C to 95°C to determine the melting temperature of the amplicons. For each amplicon, samples showing an aberrant melting curve were purified on silica filtration microplates (Whatman hydrophilic GF/C Filter, GE Healthcare Life Science, Piscataway, USA) prior to Sanger sequencing in order to identify the variation causative of the observed difference in melting curves. Sequencing was performed using Big Dye Terminator chemistry on an ABI Prism 3730xl automated sequencer from Applied Biosystems (Applied Biosystems, Foster City, CA, USA).

All sequence variants that were either unreported or had an allele frequency of <1% in the large scale reference groups “Caucasian Americans”, “African Americans” and “East Asians” based on Exome Variant Server (EVS) [31] and 1,000 genomes project (1000G) data (1000 Genomes) were confirmed by concordant variant sequencing calls of the two independent re-amplified WGA reaction products. All samples that failed either at the PCR or sequencing reaction stage were re-amplified from WGA DNAs or genomic DNAs. Samples that still did not provide satisfactory mutation screening results for at least 80% of the *ABRAXAS* coding sequence were excluded from further analysis. A total of 22 subjects were excluded from the analyses (S2 Table).

### Alignments and scoring of missense substitution

**Fig 1:**
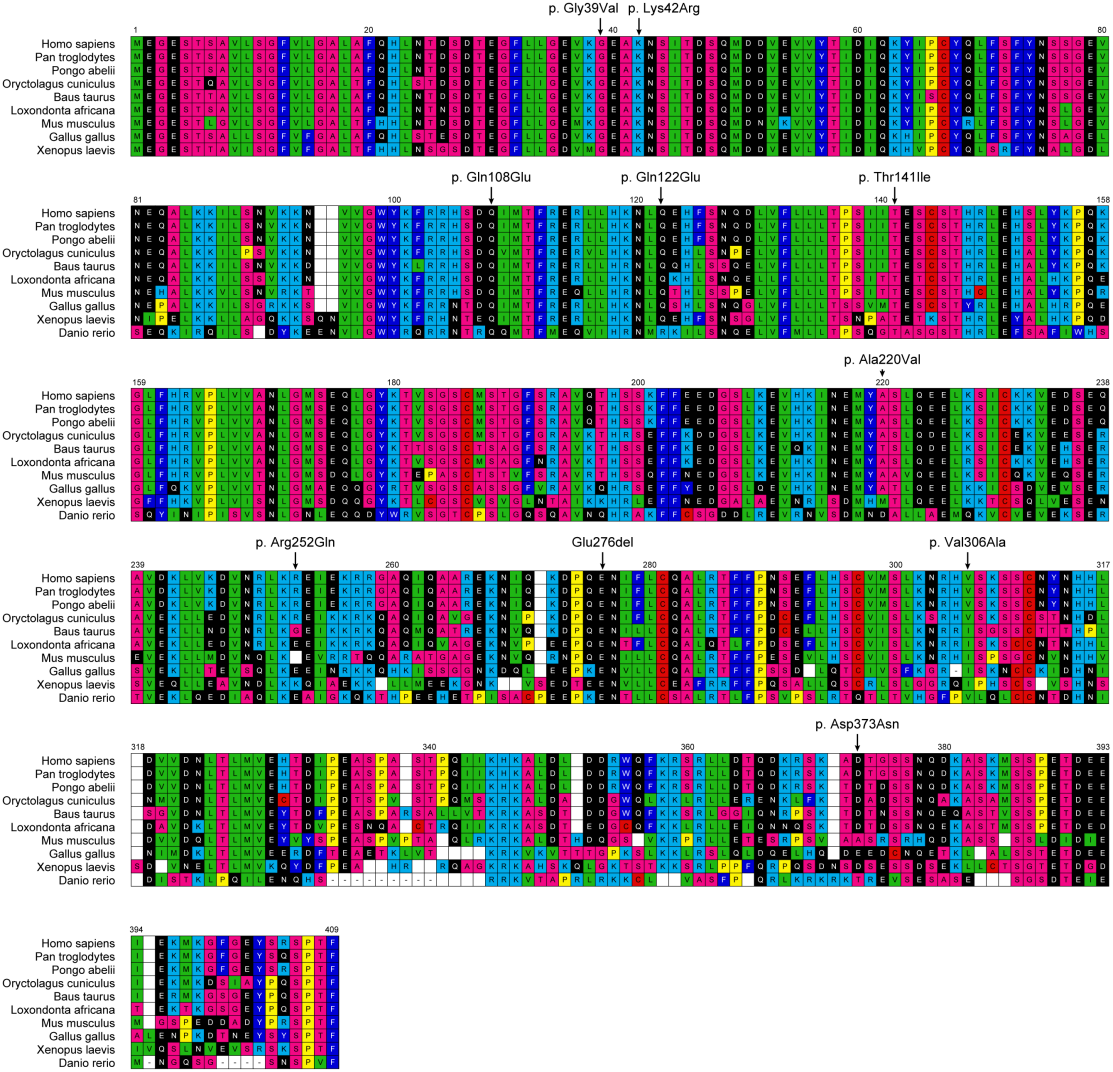
ABRAXAS multiple-sequence alignment. Substitution designations are indicated above the corresponding human reference sequence residue. Amino acid symbols are colored to represent standard Dayhoff groupings.

The multiple sequence alignment software M-Coffee, which is a part of the T-coffee software (http://tcoffee.crg.cat) (Tree-based Consistency Objective Function For alignment Evaluation) [32, 33] was used to prepare a protein multiple sequence alignment with ABRAXAS orthologs. Ten protein sequences of ABRAXAS were used, ranging from Human (*Homo sapiens*, NP_620775.2) to the most divergent zebrafish sequence (*Danio rerio*, NP_001005993.1), and including chimpanzee (*Pan troglodytes*, JAA36615.1), orangutan (*Pongo abelii*, ENSPPY00000016637), rabbit (*Oryctolagus cuniculus*, ENSOCUP00000011470), cow (*Bos taurus*, NP_0010015516.1), elephant (*Loxondonta africana*, ENSLAFP00000000891), mouse (*Mus musculus*, NP_765993), chicken (*Gallus gallus*, NP_001026315.2) and frog (*Xenopus laevis*, NP_001005339.1) (Fig 1 and S3 Table).

The impact of the missense substitutions identified during our *ABRAXAS* mutation screening was scored using our M-coffee alignment with the two prediction software programs Align-GVGD (http://agvgd.iarc.fr) and Sorting Intolerant From Tolerant (SIFT) (http://sift.jcvi.org/). PolyPhen2.1 (http://genetics.bwh.harvard.edu/pph2/) was also used with its precompiled alignment. Briefly, Align-GVGD classifies missense variants in a query sequence into 7 grades, from the most deleterious C65 to the least deleterious C0, with the intermediate grades C15, C25, C35, C45 and C55 [34]. The program is based on Grantham calculation [35], a combination of Grantham Variation (GV) which measures the amount of observed biochemical evolutionary variation at a specific position of the alignment, and Grantham Deviation (GD) which measures the biochemical difference between the missense residue and the range of variation observed at this position in the alignment. SIFT is a sequence homology-based tool that predicts variants in the query sequence as “tolerated” or “deleterious”, calculating normalized probabilities for all possible substitutions in the multiple sequence alignment. Variants with normalized probabilities <0.05 are predicted to be “deleterious” and those ≥0.05 are predicted to be “tolerated” [36]. PolyPhen2 classifies variants as “benign”, “Possibly Damaging” and “Probably Damaging”, according to eight sequenced-based and three structure-based predictive features. We used the PolyPhen2 precompiled alignment constructed and refined through a pipeline that selects homologous sequences, among which orthologs and paralogs that may or may not be full length [37].

### *In silico* analysis of variants

Intronic and exonic variants located in the vicinity of splice junction consensus sites were scored for their potential impact on splicing using the Splicing Prediction Module of Alamut (Interactive Biosoftware) which computes the following prediction methods: SpliceSiteFinder-like [38], MaxEntScan [39], NNSPLICE [40], GeneSplicer [41] and Human Splicing Finder [42]. Variants located near the beginning of the first exon were analyzed using the MatInspector software of Genomatix [43] to assess whether they altered transcription factor binding sites.

### Statistical analysis

To assess the relationship between *ABRAXAS* variants and breast cancer risk we compared frequency distribution of rare missense substitutions and in-frame deletions in cases and in controls. The case-control statistical analysis has been described in detail previously [7]. Briefly, a single table with one entry per subject, zero or one rare sequence variant per subject, annotations for type of sequence variants, study center, case-control status, and race/ethnicity was constructed. Analyses were performed using the chi square test, the Fischer test and multivariable unconditional logistic regressions using STATA version 11 software (StataCorp, College Station, TX, USA). Differences in the case/control ratio between racial/ethnic groups and study center were accounted for by including categorical variables for each racial/ethnic group and each study center. As demonstrated previously, inclusion of interactions between study center and ethnic group did not change the estimates [7]. Adjustment for racial/ethnic group should capture confounding of genetic and social factors with interaction terms, allowing that this confounding effect may be different for the broadly labeled racial/ethnic groups in different centers.

### Site-directed mutagenesis

To enable immuno-fluorescence assays for p.Gly39Val and p.Thr141Ile variants, site-directed mutagenesis was performed on a pOZ-ABRAXAS_cDNA-HA-Flag vector (kindly provided by Dr Roger A. Greenberg, University of Pennsylvania, Philadelphia, PA, USA), containing the full-length cDNA fragment encoding the complete amino acid sequence for human ABRAXAS. The vector was mutated at two positions, c.116G>T (p.Gly39Val) and c.422C>T (p.Thr141Ile) using the QuickChange Site-directed mutagenesis kit from Stratagene (Stratagene Cloning Systems, La Jolla, CA, USA) according to the manufacturer’s protocol and in conditions specific to each primer pairs for variants c.116G>T (F: 5’-TTGGGGAAGTAAAAGT**T**GAAGCCAAGAACAG-3’/R: 5’-CTGTTCTGGCTTC**A**ACTTTTACTTCCCAA-3’) and c.422C>T (F: 5’-ACCAAGTATAATAA**T**AGAAAGCTGCTCTACTC-3’/R: 5’-GTAGAGCAGCTTTCT**A**TTATTATACTTGGT-3’) respectively. DNA templates were degraded with 1 μL of DpnI at 37°C during 1 h. The resulting constructs were purified (Sigma-Aldrich Kit, St. Louis, USA) and insertion of the desired mutation was confirmed by direct sequencing of both strands using Big Dye Terminator chemistry on an ABI Prism 3730xl automated sequencer from Applied Biosystems (Applied Biosystems, Foster City, CA, USA).

### Cell culture

The Human Embryonic Kidney cell line (HEK293T) was grown in DMEM high glucose (Wisent, St-Bruno, Canada) containing 10% FBS, 2 mM L-glutamine and penicillin/streptomycin. The ER+ human breast adenocarcinoma cells, MCF7, were cultured in DMEM/F12 (Wisent Bioproducts, St-Bruno, Quebec, Canada) supplemented with 5% FBS, 2 mM L-glutamine, penicillin/streptomycin, 15 mM HEPES, 0,2% sodium bicarbonate and 10E-9 M estradiol. The human cervical carcinoma cells, HeLa, were grown in DMEM medium (Wisent) with 5% FBS, 2 mM L-glutamine and penicillin/streptomycin. Cells were grown at 37°C in a 5% CO2 incubator.

### RNA interference

Two *ABRAXAS* short hairpin RNAs (shRNA) (designated shABX139 and shABX145) cloned into the pLKO.1-puro vector were retrieved from the Sigma Mission human shRNA library available at the CHU de Quebec Research Centre (clone numbers TRCN0000139032 and TRCN0000145012). The pLKO.1-puro vector encoding a scramble sequence not matching any mammalian sequence was used as a control (designated shNSCTL). Viral supernatants were generated by transfecting HEK293T cells with the shRNA constructs and the packaging vectors psPAX2 and pMD2.G (Addgene, Cambridge, MA). The high-titer lentiviral supernatants in the presence of 10μg/ml of polybrene were used to transduce MCF7 and HeLa cell lines. Two days later, puromycin (2 μg/ml) was added to the culture media to select stably transduced cells. The expression levels of *ABRAXAS* were thereafter confirmed by quantitative PCR (see S2 Fig for knockdown efficiencies in MCF7 and HeLa cells).

### Quantitative RT-PCR

Total RNA was extracted using TriReagent (Sigma-Aldrich) following manufacturer’s instructions. Reverse transcription was performed with the M-MuLV Reverse Transcriptase (NEB) and a mix of random hexamers and anchored oligo(dT). Quantitative PCR reactions were performed using four ng of cDNA, analyzed in three replicates for each data point, in a quantitative PCR (CFX384 RealTimeSystem (Bio-Rad)) using the iTAQ Universal SYBR Green Supermix (Bio-Rad). The following forward and reverse primers were used: 5’-AGAGATAATTAAAGTTCTTGACAAAAC-3’ and 5’-TCAAATAATGGGTAAGAAAGAATAC-3’. PCR volume was 10 μl and conditions were as follow: Initial activation at 95°C, 30s followed by 40 cycles at 95°C, 5s and 60°C, 30s; final cycle (melting curve) 65°C, 0.05s and 95°C 0.5s. Relative expressions were calculated using the CFX Manager Software v3.1 using U6 and ACTB as normalizers.

### Immunofluorescence assays

Four hundred nanograms of empty pOZ-HA-Flag vector, pOZ-ABRAXAS-HA-Flag vector mutated at positions c.116G>T (p.Gly39Val), c.422C>T (p.Thr141Ile) or WT, were transfected in the two independent ABRAXAS KD MCF7 and HeLa cell lines (shABX139 and shABX145) as well as in the shNSCTL MCF7 and HeLa cell lines with Effectene transfection agent (QIAGEN, Valencia, USA). Transfected MCF7 cells were treated with 50 ng/ml of neocarzinostatin (SIGMA) and transfected HeLa cells with 25ng/ml for 30 min and released in new media for 4 hours (20 hours for empty pOZ-HA-Flag vector specifically), washed twice in PBS, fixed with 2% paraformaldehyde in PBS for 10 min, washed with TBS and fixed with cold methanol (−20°C) for 5 min. Next, cells were washed once in TBS, permeabilized 5 min with PBS (0.2% Triton X-100) and washed three times 5 min with TBS. Then, cells were quenched with 0.1% Sodium Borohydride 5 min, washed once with TBS and blocked in PBS (10% goat serum and 1% BSA) 1h. Cells were then incubated 1 h with the primary antibody diluted in 1% BSA/TBS. Cells were washed three times 5min with TBS and incubated 1 h with the appropriate secondary antibody (1% BSA/TBS) conjugated to a fluorophore (Alexa Fluor 488 (green) and Alexa Fluor 568 (red) from Life Technologies). Cells were washed three times 10 min with TBS and coverslips were mounted onto slides with PBS-glycerol (90%) containing 1 mg/ml paraphenylenediamine and 0.2 mg/ml of 4,6-diamidino-2-phenylindole (DAPI). For gamma-H2AX scoring, images were obtained using a Leica CTR 6000 microscope. Number of foci per cell was automatically counted following background subtraction and deconvolution using Volocity software v 5.5 (Perkin-Elmer Improvision). Foci were scored according to intensity within a ≤ 0.7μM radius. P-values were obtained using Wilcoxon’s Test with N = 100 cells from four independent experiments for mutant and WT pOZ-HA-Flag vectors, while N = 129 cells from three independent experiments were used to calculate p-values for empty pOZ-HA-Flag vector.

### Transient transfection and transcriptional activity assay

Constructs: To assess the impact of variant p.21G>A (rs145796091) on transcriptional activity, a fragment containing exon 1 and part of the promoter of *ABRAXAS* was amplified using genomic DNA from an individual heterozygous at this position, and subcloned into the firefly luciferase-reporter pGL3-Basic vector (Promega, Madison, WI, USA). The following primers were used for amplification (5'-**CTAGCTAGCTAG**GTGGCATATCCACTGTGGCATCGT-3′, 5′-**CCGCTCGAGCGG**AGGGCTAATGCTGGAGAAGACTTCGTGG-3′). The resulting constructs were sequenced to confirm the presence of the expected polymorphic site, amplified and then purified using Sigma GenElute HP Plasmid Kit (Sigma-Aldrich, St-Louis, MO, USA) prior to transfection. Three different clones were obtained for each genotype, in order to take into account possible inter-clone variability during luciferase reporter assays.

MCF7 cells were seeded in 24-well culture dishes at a density of 130,000 cells/well for 24 h prior to transfection. Transient transfection was performed using Lipofectamine 2000 transfection reagent (Life Technologies Inc., Ontario, Canada) according to the supplier’s protocol. Briefly, MCF7 cells were co-transfected with 1 ug of pGL3-promoter genotype-specific constructs encoding a modified firefly luciferase gene and 10 ng of a CMV-driven Renilla luciferase pRL-CMV plasmid (Promega, Madison, USA) (ratio 100:1) to control for transfection efficiency. The promoterless pGL3-basic vector and pGL3-SV40 control vector, containing the SV40 early promoter, were used as negative and positive controls, respectively. Cells were harvested 24 h post-transfection and luciferase reporter gene activities measured with the Dual-Luciferase Reporter Assay System according to the manufacturer’s instructions (Promega, Madison, USA) in a M-1000 luminometer (TECAN). The promoterless pGL3-basic vector was used to measure basal expression levels. Each experiment was performed three times. Luciferase activity was normalized to Renilla luciferase. Data from four replicates per construct were analyzed with a mixed model including the fixed effect of genotype and the random effects of experiment number and clone within experiment number. The analysis was done at the 0.05 level of significance. The model was fitted using the lme function of the nlme R package [44].

### Splicing reporter mini-gene assays: Vector construction, RNA extraction and RT-PCR

Exon 1, including the sequence variant c.21G>A (p.Ser7Ser), exon 2 and exon 3 of *ABRAXAS* were amplified separately by PCR using 50 ng of genomic DNA from an individual carrying the variant, and specific primer pairs (S4 Table). The amplified products were subcloned in a pcDNA3.1(+) vector (Invitrogen, Life Technologies, Burlington, Canada) in a three-step strategy: first, exon 1 was digested with NheI and HindIII (New England Biolabs, Beverly, MA) and inserted in the vector, then exon 2 was digested with HindIII and EcoRV and inserted in the pcDNA3.1-exon1, and finally exon 3 was inserted following its digestion with EcoRV and XhoI. The obtained pcDNA3.1-exon1-exon2-exon3 construction was transfected in HEK293T cells using Lipofectamine 2000 (Invitrogen, Life Technologies, Burlington, Canada) according to the supplier’s protocol. After 24 hours, cells were collected and total RNA was extracted using the TRI-Reagent Solution Protocol according to supplier’s instructions (Sigma-Aldrich, St Louis, USA). Reverse transcription polymerase chain reaction (RT-PCR) was performed using a PROMEGA Access RT-PCR System kit (PROMEGA, Madison, USA) according to the manufacturer’s protocol, with 50ng of the total extracted RNA and the primer pair F: 5’-ACGACTCACTATAGGGACCACAGG-3’ / R: 5’-AGAAGGCACAGTCGAGGCTGATCAG-3’ specific to the exogenous mRNA of the construction. Finally, the RT-PCR product was analyzed both by gel-electrophoresis, using 2% agarose gel, and by Sanger sequencing.

## Results

### Case-control mutation screening

A total of 2,455 subjects from the three study centers that constitute the population-based arm of the BCFR were screened for mutations. The distribution of cases and controls by race/ethnicity and study center are detailed in Table 1. In addition to the common missense substitution p.Asp373Asn (rs13125836), HRM screening revealed sixteen rare distinct sequence variants, including an in-frame deletion found in one case and one control, eight missense substitutions, four silent substitutions, two intronic variations and one variant located in the 5’-UTR region (S1 Fig). The distribution of the rare variants (minor allele frequency less than 1% in Exome Variant Server) in cases and controls is shown in Table 2.

**Table 2:** Distribution of *ABRAXAS* rare variants (*i*.*e*. with a minor allele frequency<1% in the Exome Variant Server (EVS)) identified in the BCFR.

Variant ^¶^	Effect on protein	Reference	Cases (N = 1,318)	Controls (N = 1,115)	Prediction of variant effect		
					Align-GVGD	SIFT	PolyPhen2
***Missense substitutions***							
c.116G>T	p.Gly39Val	-	1	0	C65	Damaging	Probably Damaging
c.125A>G	p.Lys42Arg	rs201948472	1	1	C0	Tolerated	Probably Damaging
c.322C>G	p.Gln108Glu	-	1	1	C25	Damaging	Possibly Damaging
c.364C>G	p.Gln122Glu	rs137876115	3*	0	C0	Tolerated	Possibly Damaging
c.422C>T	p.Thr141Ile	rs150207999	17	13	C65	Damaging	Probably Damaging
c.659C>T	p.Ala220Val	-	1	1	C0	Tolerated	Possibly Damaging
c.755G>A	p.Arg252Gln	rs114513239	3	2	C0	Tolerated	Benign
c.917T>C	p.Val306Ala	rs138986552	1	0	C25	Damaging	Benign
***In-frame deletion***							
c.826-828delAGG	p.GLu276del	-	1	1	N/A	N/A	N/A
***Silent substitutions***							
c.21G>A	p.Ser7Ser	rs145796091	17*	4*	N/A	N/A	N/A
c.33G>C	p.Ser11Ser	-	5	0	N/A	N/A	N/A
c.951C>T	p.Leu317Leu	rs79357787	2	0	N/A	N/A	N/A
c.1128T>C	p.Ser376Ser	-	1	1	N/A	N/A	N/A
***5’UTR substitution***							
c.-4T>C		rs202166386	1	2*	N/A	N/A	N/A
***Intronic variations***							
c.179-35_179-32delTAAT	-	rs199678739	31*	26*	N/A	N/A	N/A
c.681+21C>T	-	rs188169329	0	1	N/A	N/A	N/A

N/A, Not Applicable.

^**¶**^ NM_139076.2 was chosen as reference sequence.

*One Caucasian control carried both c.-4T>C and c.179-35_179-32delTAAT. One Latino control and one Latino case carried both c.21G>A and c.179-35_179-32delTAAT. One Latino case carried both c.364C>G and c.179-35_179-32delTAAT.

### Analysis of missense substitutions and in-frame indels

The potential functional impact of the missense substitutions was assessed using the three *in silico* prediction programs: Align-GVGD, SIFT and PolyPhen2. The common SNP p.Asp373Asn and the rare missense substitutions p.Lys42Arg, p.Gln122Glu, p.Ala220Val and p.Arg252Gln were predicted to be benign by at least two prediction tools, while p.Gly39Val, p.Gln108Glu, p.Thr141Ile and p.Val306Ala were predicted to be damaging or possibly damaging (Table 2). In particular, the protein multiple sequence alignment revealed complete evolutionary conservation of the ancestral amino acid sequence in the regions surrounding codon 39 and codon 141 among all species investigated (Fig 1), and p.Gly39Val and p.Thr141Ile were assigned to the most severe grade with the three algorithms.

A simple binary classification combining all rare variants affecting the coding sequence of *ABRAXAS* did not reveal any significant difference between cases and controls (*p* = 0.27). A similar result was obtained when excluding the four likely neutral variants graded C0 with Align-GVGD (Table 3). Because the missense substitution p.Thr141Ile appeared to have a frequency >1% in the European population in our study, we assessed its contribution to breast cancer risk independently and ruled out an association with the disease in the BCFR population (Table 4).

**Table 3:** Analysis of potentially pathogenic *ABRAXAS* in-frame deletion or rare missense substitutions.

Class	Cases (N)	Controls (N)	Crude OR (95% CI)	Adj^a^ OR (95% CI) (ethnicity and center)
Non-carriers	1,289	1,096		
All rare variants (incl. C0)	29	19	1.30 (0.72, 2.33) p = 0.38	1.41 (0.77, 2.57) p = 0.27
All rare variants (>C0) ^b^	21	15	1.19 (0.61, 2.32) p = 0.61	1.32 (0.67, 2.63) p = 0.42
All rare variants (incl. C0), excluding p.Thr141Ile	12	6	1.70 (0.64, 4.55) p = 0.29	1.61 (0.58, 4.47) p = 0.36

^a^ OR are adjusted for race or ethnicity (Caucasian, East Asian, African American or Latina) and study center.

^b^ In the binary analysis, only carriers of a missense substitution with grade>C0 or of an in-frame deletion (IFR) were considered.

**Table 4:** Distribution of p.Thr141Ile, p.Ser7Ser and p.Ser11Ser by race/ethnicity.

Variant	Race/Ethnicity	Cases	Controls	Trend test p-value (crude analysis)	Trend test p-value (adjusted analysis)^a^
p.Thr141Ile	All	17/1301	13/1102	0.78	0.49
	European	13/844	13/949	0.77	0.76
	Latino	2/151	0/47	*-*	-
	East Asian	1/209	0/71	-	-
	African-American ancestry	1/97	0/35	-	-
p.Ser7Ser	All	17/1301	4/1111	0.021	0.61
	European	0/857	0/962	-	-
	Latino	12/141	4/43	0.88	0.90
	East Asian	5/205	0/71	(0.33)*	-
	African-American ancestry	0/98	0/35	-	-
p.Ser11Ser	All	5/1313	0/1115	(0.07)*	-
	European	0/857	0/962	-	-
	Latino	0/153	0/47	-	-
	East Asian	5/205	0/71	(0.33)*	-
	African-American ancestry	0/98	0/35	-	-

*Fisher’s exact test p-value

^a^ OR are adjusted for study center (and for race or ethnicity in the combined analysis).

We also analyzed independently the common missense substitution p.Asp373Asn (rs13125836) in this BCFR series. The observed minor allele (A) frequency in the four racial/ethnic groups represented is shown in Table 5. No departure from Hardy-Weinberg equilibrium was observed in either cases or controls. Overall, there was no significant difference in allele frequencies between cases and controls for this SNP when pooling the different populations or after stratifying by race/ethnicity.

**Table 5:** Stratified analyses of the common SNP rs13125836 (c. 1117G>A, p.Asp373Asn) on breast cancer risk in the BCFR.

	Number of genotyped subjects Cases / Controls	A allele frequency Cases / Controls	Chi^2^ *P*-value^a^	Log-additive model^b^ OR* [95% CI]	*P*-trend
**All**	1,318 / 1,115	0.038 / 0.036	0.70	0.90 [0.65, 1.24]	0.50
**By race/ethnicity**					
European	857 / 962	0.035 / 0.040	0.48	0.85 [0.59, 1.21]	0.36
East Asian	210 / 71	0.017 / 0.0	0.12	-	-
Recent African ancestry	98/ 35	0.066 / 0.071	0.88	0.92 [0.30, 2.79]	0.88
Latina	153 / 47	0.049 / 0.043	0.80	0.85 [0.26, 2.83]	0.80

^a^Test for the difference in A allele frequency between cases and controls.

^b^Results of the logistic regression assuming a log-additive model with study center included in the regression model as covariate in the combined analysis, and with race/ethnicity and study center as covariates in the stratified analysis (*OR is given for heterozygous carriers of the A allele).

### Immunofluorescence assays of ABRAXAS missense substitutions graded damaging by Align-GVGD, SIFT and PolyPhen2 algorithms

To assess the impact of the two variants classified as damaging (C65; Align GVGD) for protein function (p.Thr141Ile and p.Gly39Val), formation of repair foci was analyzed by immunofluorescence (IF) assays. To do this, we first generated MCF7 and HeLa *ABRAXAS* knockdown cells using two different shRNAs (see S2 Fig for expression knockdown efficiencies) to evaluate whether the knock down of ABRAXAS had a functional consequence. Using staining of gamma-H2AX as a marker for damaged DNA, we found that shABRAXAS cell lines (shABX139 and shABX145) showed a higher percentage of cells containing gamma-H2AX foci, compared to shNSCTL, 20 hrs post-treatment with the DSB-generating agent neocarzinostatin, indicative of a deficiency in DNA repair (S3 Fig). A significant difference is observed between both shABRAXAS clones and the shNSCTL in HeLa cells (p-values for shABX139 and shABX145 are 0.0005 and 0.0065, respectively). In the MCF7 cell line, a significant difference is observed for shABX145 (p-value = 0.00167) while an increase in the percentage of cells containing gamma-H2AX foci is also observed for shABX139, albeit this increase is not significant (p-value = 0.2145). Although these results appear to show that the partial knock down of ABRAXAS was sufficient to result in a functional consequence, these results should be interpreted with caution.

**Fig 2:**
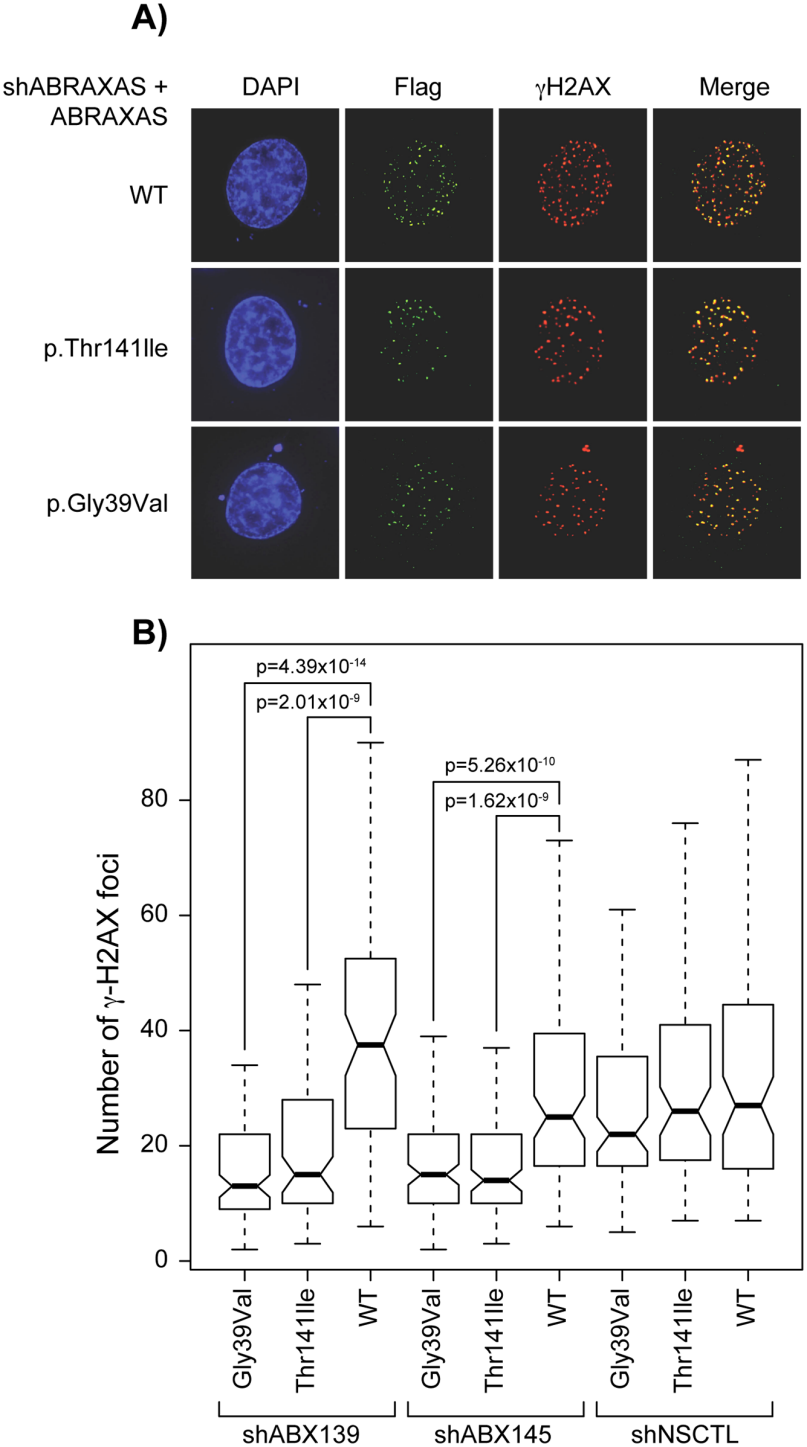
p.Gly39val and p.Thr141Ile ABRAXAS mutants have defects in gamma-H2AX formation. (A) Typical DNA damage foci of ABRAXAS in shABRAXAS (shABX145) MCF7 cells complemented with ABRAXAS-HA-Flag, ABRAXAS-HA-Flag pThr141Ile, or ABRAXAS-HA-Flag pGly39Val. The anti-Flag antibody was used to monitor ABRAXAS foci formation (green), anti-gamma-H2AX (red) and the merge picture is depicted. In blue, DAPI staining. (B) Quantification of gamma-H2AX foci formation in MCF7 cells after neocarzinostatin treatment and release. P-values were obtained with a Wilcoxon’s Test with N = 100 cells from four independent experiments.

Thereafter, shABRAXAS cells were complemented with ABRAXAS-HA-Flag, ABRAXAS-HA-Flag p.Thr141Ile, or ABRAXAS-HA-Flag pGly39Val, to assess the impact of the two variants on DNA repair. As illustrated in Fig 2A, IF of wild-type, p.Thr141Ile and p.Gly39Val ABRAXAS show that DNA repair foci are formed even in the presence of the variants, indicating that these variants do not negatively impact the recruitment of the A-complex to DNA damage sites. Next, we monitored the formation of gamma-H2AX foci after treatment with neocarzinostatin. After a 4h release, gamma-H2AX foci formation was significantly reduced in both shABRAXAS MCF7 cell lines (shABX139 and shABX145) transfected with p.Thr141Ile (p-value 2.01x10^-9^ for shABX139 and 1.62x10^-9^ for shABX145) and p.Gly39Val (p-value 4.39x10^-14^ for shABX139 and 5.26x10^-10^ for shABX145) compared to WT. On the other hand, we did not observe a significant difference between the non-silencing control cell line transfected with either the WT or the mutant constructs. Thus the significant difference in foci distribution observed in shABRAXAS MCF7 cell lines between variants p.Thr141Ile and p.Gly39Val and WT (Fig 2B) suggests that the two variants either: 1) impair formation of DNA repair foci or 2) impair DNA damage signaling after DSB formation. Experiments were also performed in the HeLa cell line (S4 Fig). Results did not show significant differences in the formation of gamma-H2AX foci between WT and p.Gly39Val in both shABRAXAS knock down cell lines (p-values 0.085 and 0.081 for clones shABX139 and shABX145 respectively), while a significant decrease was observed for p.Thr141Ile, albeit this difference being weaker than that observed in MCF7 cells (p-values 0.001 and 0.0005 for clones shABX139 and shABX145 respectively, compared to p = 2x10^-9^ and p = 1.6x10^-9^ in MCF7).

### Analysis of silent substitutions, 5’-UTR and intronic variations

**Fig 3:**
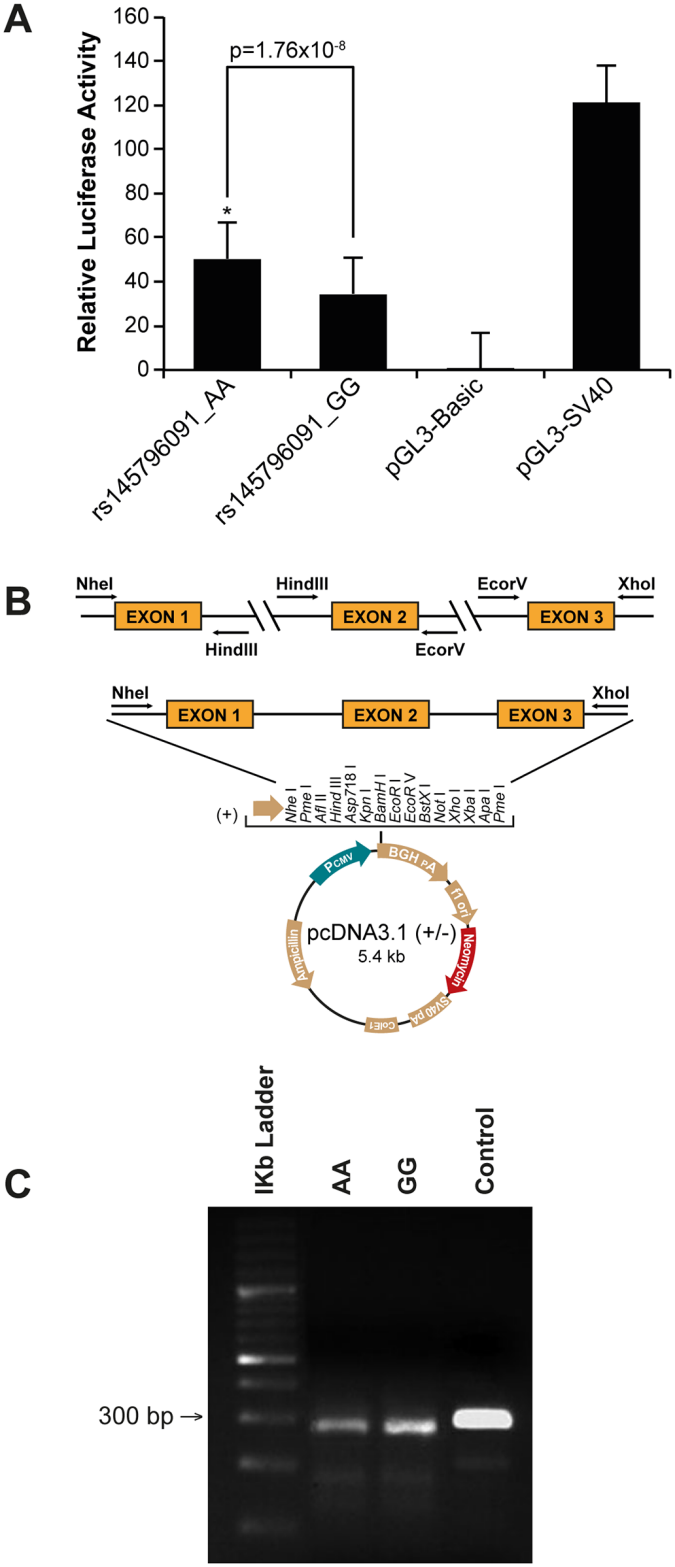
Functional assays assessing the impact of variant rs145796091 c.21G>A (p.Ser7Ser) on transcriptional activity and splicing efficiency in the MCF7 breast cancer cell line. A) Gene reporter assays. Luciferase activity was normalized to Renilla luciferase. Each experiment was performed three times. Data from four replicates per construct were analyzed with a mixed model including the fixed effect of genotype and the random effects of experiment number and clone within experiment number. B) Splicing reporter mini-gene assays. Mini-gene constructions: exons 1, 2 and 3 of *ABRAXAS* were subcloned in a pcDNA3.1 vector. C) Gel-electrophoresis of mini-gene RT-PCR products on 2% agarose gels.

The variant c.-4T>C located in the 5’-UTR of *ABRAXAS* and the two silent substitutions c.21G>A (p.Ser7Ser) and c.33G>C (p.Ser11Ser) located near the beginning of the first exon were analyzed using the MatInspector software of Genomatix [43] in order to predict whether they altered transcription factor consensus binding sites. These analyses showed that the variant c.21G>A (p.Ser7Ser) abolished a consensus binding site for the Early Growth Response (EGR2) transcription factor, while the two other examined variants were not predicted to affect the binding sites of any transcription factors known to be involved in breast cancer. Gene reporter assays were performed in the breast cancer cell line MCF7 using a promoter fragment containing c.21G>A (p.Ser7Ser) to assess the impact of the variant on transcriptional activity. A 1.4 fold increase in transcriptional levels was observed for the construct containing the variant compared to the wild-type construct (F_(1,48) = 45.6, p = 1.76 x 10^−8^) (Fig 3A). Due to the proximity of c.21G>A (p.Ser7Ser) variant to the beginning of the first exon, we also investigated whether this variant could alter the splicing of intron 1 using the *in silico* prediction program Human Splicing Finder [36]. Predictions using this program revealed that the variant disrupted the consensus binding site for hRNP8G9, a protein involved in the splicing machinery. In attempt to confirm this, splicing reporter mini-gene assays were used to evaluate the effect of this variant on splicing between exons 1 and 2 (Fig 3B). After transient transfection in HEK293T cells, the splicing patterns of the transcripts generated from the wild-type and c.21G>A variant constructs were compared by reverse transcription-PCR analysis and direct sequencing (Fig 3C). No differences in splicing patterns were observed suggesting that the variant has no impact on the splicing between exon 1, exon 2 and exon 3.

*In silico* analysis of the intronic variants c.681+21C>T and c.179-35_179-32delTAAT did not reveal any predicted impact on splice junction consensus sites.

## Discussion

To our knowledge, this study is the largest case-control mutation screening investigating whether rare sequence variations within *ABRAXAS* contribute to breast cancer susceptibility. This gene was an appealing candidate because of (i) its central role in the formation of the A-complex and it’s critical functions during HR repair, (ii) its implication in BRCA1 recruitment to DNA double-strand breaks, (iii) its interaction with the proteins encoded by the two known breast cancer susceptibility genes *BRCA1* and *BABAM1 (MERIT40)* and (iv) its identification as a breast cancer susceptibility gene in Finnish breast cancer families.

Our mutation screening identified 17 *ABRAXAS* variants: 11 were already reported in the public databases such as EVS (Exome Variant Server) [31] and 1,000G (1000 Genomes) and 6 are novel variants. While the two missense variants, p.Thr141Ile (rs150207999) [45] and p.Asp373Asn (rs13125836) [27, 45] were previously reported in mutation screening studies performed in Spanish and Finnish high-risk breast cancer families, their respective frequencies in cases and controls in the previously mentioned studies [27, 45] did not suggest a role of these alleles in breast cancer susceptibility. Similarly, the frequencies of these variants in our study were not indicative of significant increased risk of breast cancer. However, *in silico* analyses using the three prediction tools Align-GVGD, SIFT and PolyPhen2 have classified the p.Thr141Ile variant at the highest grades of damage for the protein and this variant is located in the RAP80-binding domain. RAP80 recognizes the ubiquitinated proteins at sites of DNA breaks, which in turn facilitates the recruitment of BRCA1 to DNA double strand breaks via a direct interaction between BRCA1 and ABRAXAS [23, 46, 47]. Thus, an alteration of the sequence in this binding domain could impair the localization of RAP80 and other binding partners at sites where DNA repair is needed.

The second variant predicted to be deleterious to the protein, p.Gly39Val, was not previously reported and was only observed in one African-American case, therefore preventing the analysis of any association with breast cancer risk.

We thus investigated through immunofluorescence assays whether the two variants predicted to be most damaging, p.Thr141Ile and p.Gly39Val, had a functional impact on the protein (Fig 2)., In MCF7 shABRAXAS cells complemented with the variants, both variants localized to DNA damage sites, however the number of gamma-H2AX foci formed was significantly different from WT complemented shABRAXAS cells. Taken together, these results suggest that these variants affect the DNA damage response and are thus likely to play a role in breast cancer susceptibility, but much larger studies would be required to test this hypothesis due to the rarity of the two variants. The decrease of gamma-H2AX phosphorylation could be explained by several means including defective activation of ATM/ATR. Further work is warranted to confirm the functional impact of these variants on ABRAXAS.

Solyom *et al* provided evidence that a mutation in *ABRAXAS* could indeed have a significant impact of the proper functioning of the DNA repair pathway. It has recently been reported that the mutation c.1082G>A (p.Arg361Gln), identified in 2.4% of the 125 Finnish high-risk breast cancer families (*P* = 0.002 –familial cases versus controls), which impaired the localization of the protein in cultured cells, caused hypersensitivity to IR and reduced BRCA1 localization at sites of DNA damage [27]. This variant was not observed in our BCFR study population which was ascertained in North America and Australia, and included Caucasians of European Ancestry, Latinas and African-Americans suggesting that this variation may be specific to the Finnish population.

Analysis of silent and intronic substitutions did not reveal significant associations of these variants with breast cancer risk (Table 2). Interestingly, c.21G>A; p.Ser7Ser and c.33G>C; p.Ser11Ser were observed only in Latinas and East Asians (Table 4). The *in silico* analyses and experimental assays performed in this study did not support a functional role for these variants. Over-representation of these variants in these specific populations would warrant further investigation in these racial/ethnic groups using larger sample sizes, in order to provide statistical power to robustly detect an association with the disease.

Although the results of our study did not provide evidence that rare variants in *ABRAXAS* are associated with increased breast cancer risk in the populations studied, we cannot rule out the possibility that rare mutations in *ABRAXAS* may be involved in some high-risk families with more specific phenotypes. Solyom *et al*. reported that in addition to breast cancer, the families with p.Arg361Gln displayed some relatively rare types of cancer, such as lung and lip cancer and lymphoma of the throat [27]. Another study, reporting the sequencing of several homologous recombination genes in 390 ovarian carcinomas, identified the *ABRAXAS* germline mutation, c.1106insG, in 2 subjects, representing 2% of identified deleterious mutations [28]. Moreover, a recent genome-wide association study associated a common SNP (rs1494961) located downstream of the *ABRAXAS* gene with upper aero-digestive tract cancer risk (OR = 1.12, 95% CI = 1.08–1.17, p = 1 x 10–8) [48]. In view of this, much larger case-control studies are needed to determine whether allelic variants in this gene could be associated with a low or modest risk of breast cancer.

## Supporting Information

S1 Fig Mutation screening of *ABRAXAS* by High Resolution Melting curve analysis. Representative melting curves obtained from 384 samples, for exons where variants were observed. Panels (A) and (B) First exon, (C) Exon 2, (D) Exon 3, (E) and (F) Exon 5, (G) Exon 7, (H) Exon 8, (I) to (L) Exon 9.
(TIFF)

S2 Fig Quantitation of ABRAXAS mRNA levels in ABRAXAS knockdown MCF7 and HeLa cells. Quantitative PCR analysis of ABRAXAS mRNA levels in MCF7 and HeLa cells stably expressing two independent shRNAs targeting ABRAXAS (shABX139 and shABX145) and a control shRNA containing a non-target shRNA (shNSCTL). ACTB (beta-actin) and U6 snRNA were used as endogenous controls for normalization.
(PPTX)

S3 Fig Quantification of gamma-H2AX formation in shABRAXAS (shABX139 and shABX145) and shNSCTL A) MCF7 and B) HeLa cells complemented with empty pOZ-HA-Flag vector after neocarzinostatin treatment and release. P-values were obtained with a Wilcoxon’s Test with N = 129 cells from three independent experiments.
(TIF)

S4 Fig Quantification of gamma-H2AX formation in shABRAXAS (shABX139 and shABX145) HeLa cells complemented with ABRAXAS-HA-Flag, ABRAXAS-HA-Flag pThr141Ile, or ABRAXAS-HA-Flag pGly39Val after neocarzinostatin treatment and release. P-values were obtained with a Wilcoxon’s Test with N = 100 cells from four independent experiments.
(TIF)

S1 Table Primers used for High Resolution Melting amplification. (DOC)

S2 Table Subjects excluded because of poor mutation screening performance, by study center. (DOC)

S3 Table ABRAXAS protein multiple sequence alignment characterization. (DOC)

S4 Table Primers used for subcloning exons 1, 2 and 3 into p.cDNA3.1 (+). (DOC)
