# Supplementary figures and images for: ABRAXAS (FAM175A) and Breast Cancer Susceptibility: No Evidence of Association in the Breast Cancer Family Registry
Source: PLoS One. 2016 Jun 7;11(6):e0156820. doi: 10.1371/journal.pone.0156820 (PMC4896418; doi:10.1371/journal.pone.0156820)

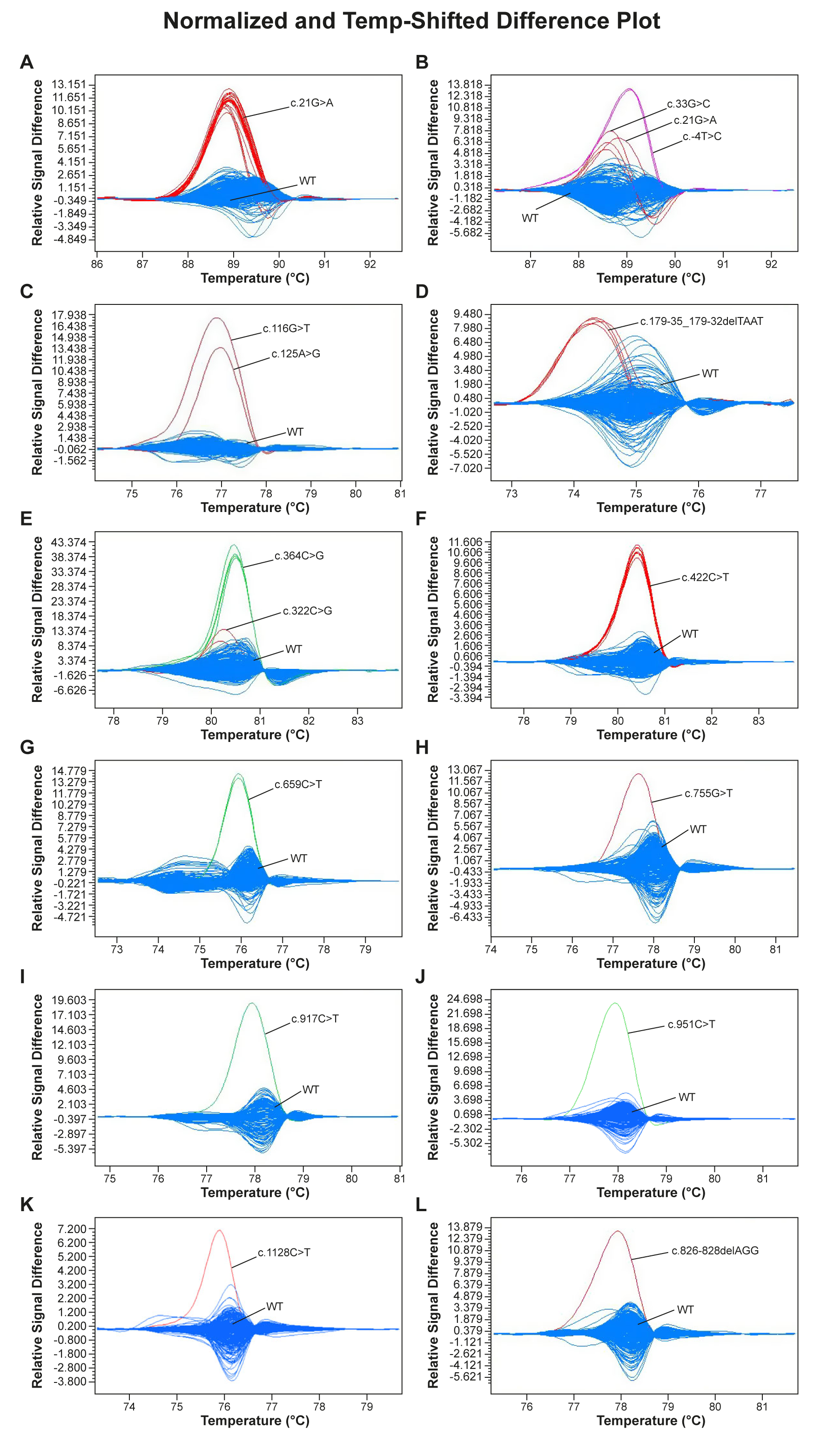

Supplement: S1 Fig — Representative melting curves obtained from 384 samples, for exons where variants were observed. Panels (A) and (B) First exon, (C) Exon 2, (D) Exon 3, (E) and (F) Exon 5, (G) Exon 7, (H) Exon 8, (I) to (L) Exon 9. (TIFF) [file pone.0156820.s001.tiff]

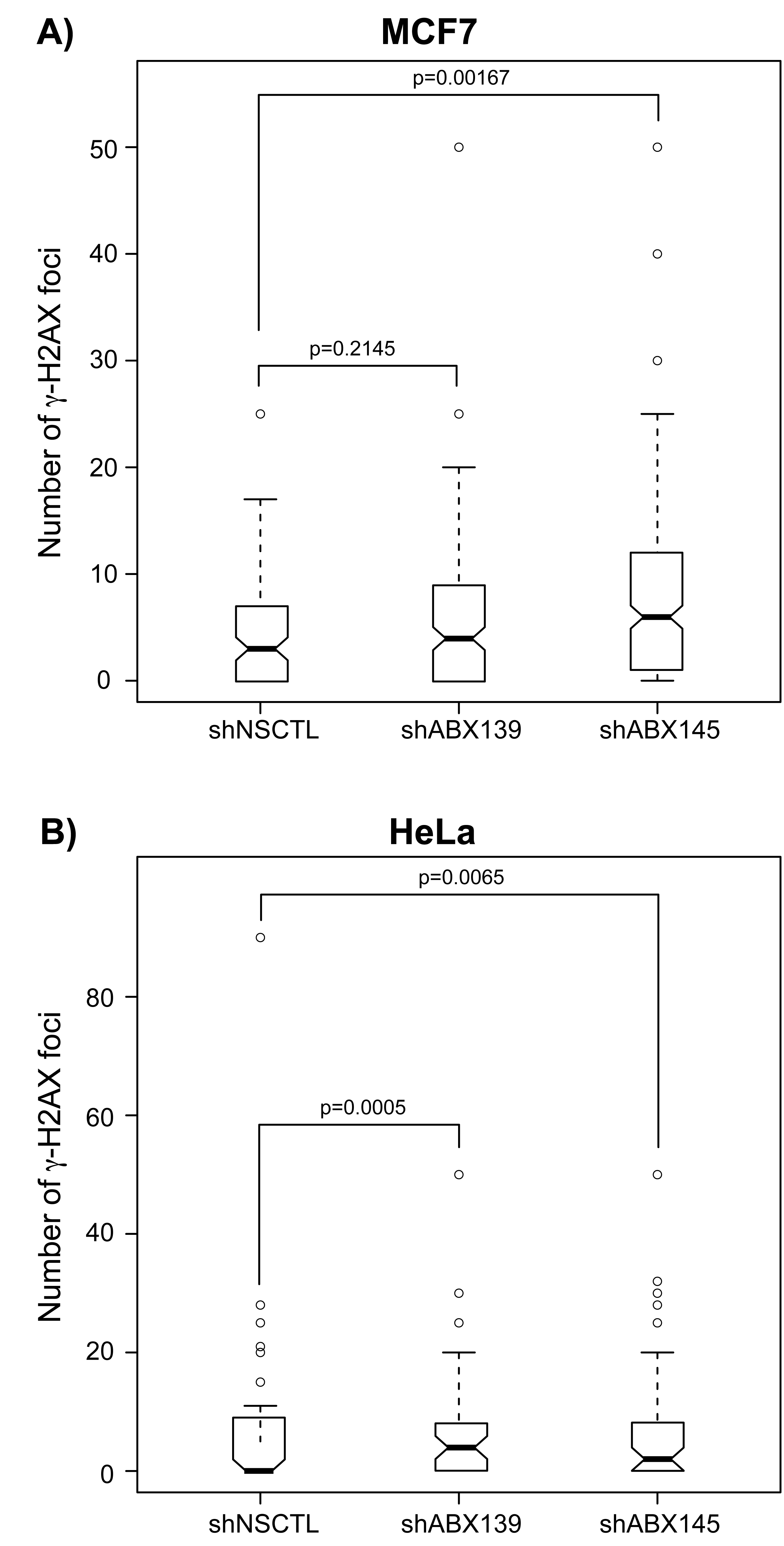

Supplement: S3 Fig — P-values were obtained with a Wilcoxon’s Test with N = 129 cells from three independent experiments. (TIF) [file pone.0156820.s003.tif]

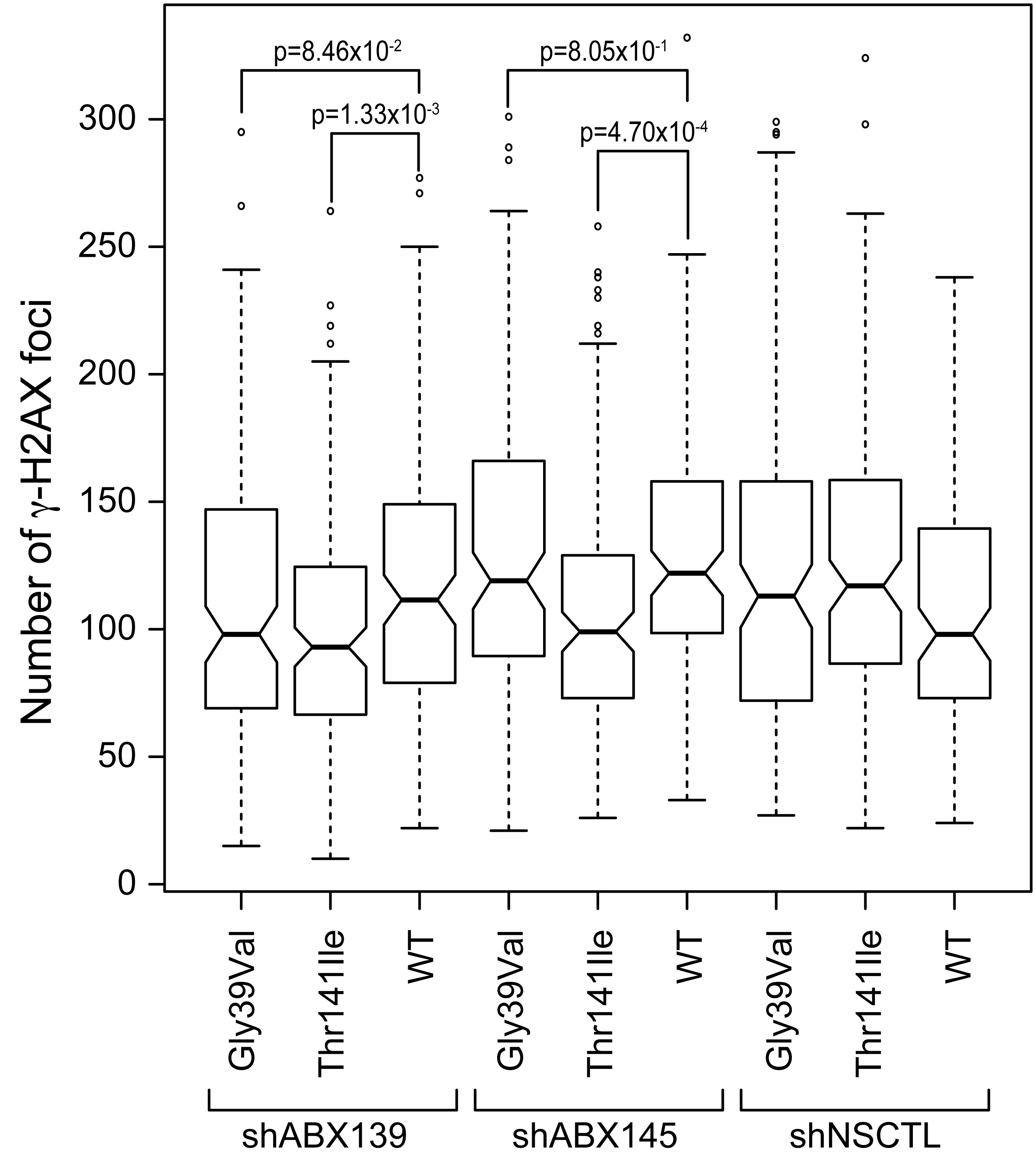

Supplement: S4 Fig — P-values were obtained with a Wilcoxon’s Test with N = 100 cells from four independent experiments. (TIF) [file pone.0156820.s004.tif]
