# Supplementary material for: ABRAXAS (FAM175A) and Breast Cancer Susceptibility: No Evidence of Association in the Breast Cancer Family Registry
Source: PLoS One. 2016 Jun 7;11(6):e0156820. doi: 10.1371/journal.pone.0156820 (PMC4896418; doi:10.1371/journal.pone.0156820)
Supplement: S2 Fig — Quantitative PCR analysis of ABRAXAS mRNA levels in MCF7 and HeLa cells stably expressing two independent shRNAs targeting ABRAXAS (shABX139 and shABX145) and a control shRNA containing a non-target shRNA (shNSCTL). ACTB (beta-actin) and U6 snRNA were used as endogenous controls for normalization. (PPTX) [file pone.0156820.s002.pptx]

## Slide 1
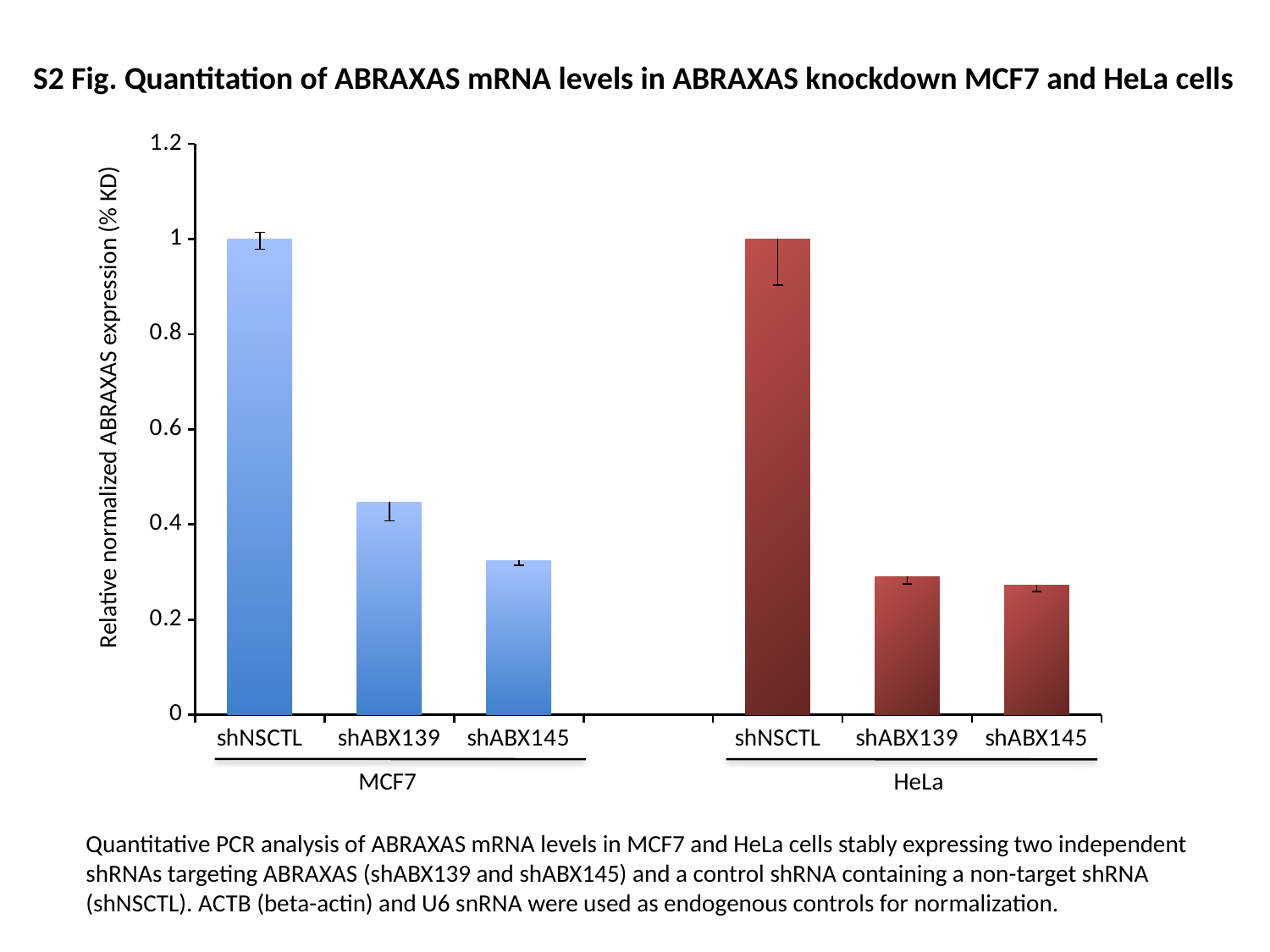

S2 Fig. Quantitation of ABRAXAS mRNA levels in ABRAXAS knockdown MCF7 and HeLa cells
### Chart
| Category | Relative normalized ABRAXAS expression |
|---|---|
| shNSCTL | 1.0 |
| shABX139 | 0.44759 |
| shABX145 | 0.32462 |
| | None |
| shNSCTL | 1.0 |
| shABX139 | 0.29118 |
| shABX145 | 0.27332 |Relative normalized ABRAXAS expression (% KD)
MCF7
HeLa
Quantitative PCR analysis of ABRAXAS mRNA levels in MCF7 and HeLa cells stably expressing two independent shRNAs targeting ABRAXAS (shABX139 and shABX145) and a control shRNA containing a non-target shRNA (shNSCTL). ACTB (beta-actin) and U6 snRNA were used as endogenous controls for normalization.
