# Supplementary material for: ABRAXAS (FAM175A) and Breast Cancer Susceptibility: No Evidence of Association in the Breast Cancer Family Registry
Source: PLoS One. 2016 Jun 7;11(6):e0156820. doi: 10.1371/journal.pone.0156820 (PMC4896418; doi:10.1371/journal.pone.0156820)
Supplement: S1 Table — (DOC) [file pone.0156820.s005.doc]

**S1 Table: Primers used for High Resolution Melting amplification**

| **EXON** | **PRIMERS (5'🡪3')** | **AMPLICON LENGTH**  **(bp)** | **ANNEALING TEMPERATURE (°C)** |
| --- | --- | --- | --- |
|  |  |  |  |
| 1 | F: TCGTCCTCTTGTGTAGCCTGAGG | 122 | 65 |
|  | R: ACCGTGTCCGAGTCCGTGTTG |
|  |  |  |  |
| 2 | F: TTCTATCAGTGTATTCTATATATTGCC | 191 | 55 |
|  | R: AAACTATCAAATATAGGAGACACAG |
|  |  |  |  |
| 3 | F: TGCAGCATTCAGGTGATTCTG | 133 | 60 |
|  | R: TGGATCATTTACTTACTAGCACTACT |
|  |  |  |  |
| 4 | F: ACCTTTAATTTTGGCTTTTACAGC | 220 | 58 |
|  | R: TTCTTACTTACTTTACAGGATACTATG |
|  |  |  |  |
| 5 | F: AGCCAGTGACTGGCCAACATTTAC | 228 | 60 |
|  | R: TGTATTTCAGAATGTGGTAGGTTGG |
|  |  |  |  |
| 6 | F: TCTTTTCATTCTAAGTTTATACTCTAA | 204 | 55 |
|  | R: AAGATGAATAGAAGTTTTGTGAGAA |
|  |  |  |  |
| 7 | F: AAGACTCTTCTATTGGTCCTTGAC | 248 | 58 |
|  | R: TGTCAGTCATTAACTTGATATGAAC |
|  |  |  |  |
| 8 | F: AGTACACAGCTTGAAATATTACCTATG | 196 | 58 |
|  | R: ACAGAAAGTAGAGATGTGTTGTTTAC |
|  |  |  |  |
| 9A * | F: TGTAGAAGAAATGTTATATGTTGAACTG | 252 | 58 |
|  | R: TTCAGGAATGTCAGTGTGTTCTAC |
|  |  |  |  |
| 9B * | F: AGTAGCTGTAACTACAACCACCAT | 190 | 62 |
|  | R: CTTTAGATCGTTTGTCTTGTGTATCT |
|  |  |  |  |
| 9C * | F: GATGACAGATGGCAATTCAAGAGATCT | 249 | 56 |
|  | R: AGTAAAAACAATAGAAATGTTTGG |
|  |  |  |  |
| Promoter | F: AACCAGCCTGGCCAACATGGTGAAAC | 210 | 64 |
| region | R:TTGAGACTGAGTCTCGCTTGGTCA |

* Exon 9 was divided into 3 parts in order to obtain amplicons ≤ 250bp
