# Supplementary material for: ABRAXAS (FAM175A) and Breast Cancer Susceptibility: No Evidence of Association in the Breast Cancer Family Registry
Source: PLoS One. 2016 Jun 7;11(6):e0156820. doi: 10.1371/journal.pone.0156820 (PMC4896418; doi:10.1371/journal.pone.0156820)
Supplement: S2 Table — (DOC) [file pone.0156820.s006.doc]

**S2 Table: Subjects excluded because of poor mutation screening performance, by study center.**

| **Study Center** | **Cases, n (%)** | **Controls, n (%)** |
| --- | --- | --- |
| BCFR-Australia | 4 (0.67%) | 7 (1.34%) |
| BCFR-Ontario | 2 (0.64%) | 0 (0.00%) |
| BCFR-Northern California | 8 (1.09%) | 1 (0.74%) |
| **Total** | 14 (1.05%) | 8 (0.71%) |
