# Supplementary material for: ABRAXAS (FAM175A) and Breast Cancer Susceptibility: No Evidence of Association in the Breast Cancer Family Registry
Source: PLoS One. 2016 Jun 7;11(6):e0156820. doi: 10.1371/journal.pone.0156820 (PMC4896418; doi:10.1371/journal.pone.0156820)
Supplement: S3 Table — (DOC) [file pone.0156820.s007.doc]

**S3 Table: ABRAXAS protein multiple sequence alignment characterization.**

| **Sequence source** | **Sequence length** | **Av. number of substitutions per position** | **SIFT: median sequence conservation score** | **Percent amino acid sequence identity in pairwise comparison** | | | | | | | | | |
| --- | --- | --- | --- | --- | --- | --- | --- | --- | --- | --- | --- | --- | --- |
|  |  |  |  | Hsap | Ptro | Pabe | Ocun | Btau | Lafr | Mmus | Ggal | Xlae | Drer |
| Hsap | 333 | N/A | N/A | 1.00 |  |  |  |  |  |  |  |  |  |
| Ptro | 333 | 0.02 | 4.32 | 0.99 | 1.00 |  |  |  |  |  |  |  |  |
| Pabe | 333 | 0.03 | 4.32 | 1.00 | 0.99 | 1.00 |  |  |  |  |  |  |  |
| Ocun | 333 | 0.63 | 4.32 | 0.82 | 0.82 | 0.83 | 1.00 |  |  |  |  |  |  |
| Btau | 334 | 0.81 | 4.32 | 0.81 | 0.81 | 0.82 | 0.81 | 1.00 |  |  |  |  |  |
| Lafr | 333 | 0.93 | 4.32 | 0.82 | 0.82 | 0.82 | 0.81 | 0.82 | 1.00 |  |  |  |  |
| Mmus | 331 | 0.48 | 4.32 | 0.72 | 0.72 | 0.72 | 0.70 | 0.71 | 0.71 | 1.00 |  |  |  |
| Ggal | 329 | 1.50 | 3.89 | 0.63 | 0.63 | 0.63 | 0.64 | 0.62 | 0.67 | 0.59 | 1.00 |  |  |
| Xlae | 332 | 2.12 | 3.69 | 0.54 | 0.54 | 0.54 | 0.53 | 0.55 | 0.56 | 0.51 | 0.58 | 1.00 |  |
| Drer | 315 | 3.05 | 3.38 | 0.38 | 0.38 | 0.38 | 0.37 | 0.38 | 0.39 | 0.36 | 0.41 | 0.37 | 1.00 |

N/A, Not applicable

Species abbreviations: Hsap, Homo sapiens (human); Ptro, Pan troglodytes (chimpanzee); Pabe, Pongo abelii (orangutan); Mmus, Mus musculus (mouse); Ocun, Oryctolagus cuniculus (rabbit); Btau, Bos taurus (cow); Lafr, Loxondonta africana (elephant); Ggal, Gallus gallus (chicken); Xlae, Xenopus laevis (frog); Drer, Danio rerio (zebrafish).
