# Supplementary material for: ABRAXAS (FAM175A) and Breast Cancer Susceptibility: No Evidence of Association in the Breast Cancer Family Registry
Source: PLoS One. 2016 Jun 7;11(6):e0156820. doi: 10.1371/journal.pone.0156820 (PMC4896418; doi:10.1371/journal.pone.0156820)
Supplement: S4 Table — (DOC) [file pone.0156820.s008.doc]

S4 Table: Primers used for subcloning exons 1, 2 and 3 into p.cDNA3.1 (+)

| **Exon** | **Restriction site** | **Primer (5’ 🡪 3’)** |
| --- | --- | --- |
|  |  |  |
| 1 | Nhe I | F: CTA**GCTAGC**TAGTGCATGTTTGTGACAGGTCGCTG |
|  | HindIII | R: CCC**AAGCTT**GAGGGCTAATGCTGGAGAAGACTTCGTGG |
|  |  |  |
| 2 | HindIII | F: CCC**AAGCTT**GGGACTCTGATTATTTCAGGCTAGAC |
|  | EcoRV | R: CG**GATATC**CGTGCAAAATGAGTACAACATATCC |
|  |  |  |
| 3 | EcoRV | F: CG**GATATC**CGGTTTATTAATTGGGCACATTTAGAGCA |
|  | XhoI | R: CCG**CTCGAG**CGGTCTACTCAGTACCACCATGTATAG |
